# Pioglitazone and bladder cancer risk: a systematic review and meta‐analysis

**DOI:** 10.1002/cam4.1354

**Published:** 2018-02-24

**Authors:** Huilin Tang, Weilong Shi, Shuangshuang Fu, Tiansheng Wang, Suodi Zhai, Yiqing Song, Jiali Han

**Affiliations:** ^1^ Department of Epidemiology Richard M. Fairbanks School of Public Health Indiana University Indianapolis Indiana; ^2^ Center for Pharmacoepidemiology Richard M. Fairbanks School of Public Health Indiana University Indianapolis Indiana; ^3^ Department of Pharmacy Peking University Third Hospital Beijing China; ^4^ School of Public Health The University of Texas Health Science Center at Houston Houston Texas; ^5^ Department of Epidemiology Gillings School of Global Public Health University of North Carolina at Chapel Hill Chapel Hill North Carolina; ^6^ Melvin and Bren Simon Cancer Center Indiana University Indianapolis Indiana

**Keywords:** Bladder cancer, dose–response relationship, meta‐analysis, pioglitazone

## Abstract

Current evidence about the association between pioglitazone and bladder cancer risk remains conflict. We aimed to assess the risk of bladder cancer associated with the use of pioglitazone and identify modifiers that affect the results. We systematically searched PubMed, Embase, and Cochrane Central Register of Controlled Trials from inception to 25 August 2016 for randomized controlled trials (RCTs) and observational studies that evaluated the association between pioglitazone and bladder cancer risk. Conventional and cumulative meta‐analyses were used to calculate the odds ratio (OR) with 95% confidence interval (CI). A restricted spline regression analysis was used to examine the dose–response relationship with a generalized least‐squares trend test. We included two RCTs involving 9114 patients and 20 observational studies (*n* = 4,846,088 individuals). An increased risk of bladder cancer in patients treated with pioglitazone versus placebo was noted from RCTs (OR, 1.84; 95%CI, 0.99 to 3.42). In observational studies, the increased risk of bladder cancer was slight but significant among ever‐users of pioglitazone versus never‐users (OR, 1.13; 95%CI, 1.03 to 1.25), which appeared to be both time‐ (*P* = 0.003) and dose‐dependent (*P* = 0.05). In addition, we observed the association differed by region of studies (Europe, United States, or Asia) or source of funding (sponsored by industry or not). Current evidence suggests that pioglitazone may increase the risk of bladder cancer, possibly in a dose‐ and time‐dependent manner. Patients with long‐term and high‐dose exposure to pioglitazone should be monitored regularly for signs of bladder cancer.

## Introduction

Pioglitazone, an agonist of peroxisome proliferator‐activated receptor *γ* (PPAR *γ*), has been shown to improve glycemic levels in patients with type 2 diabetes mellitus (T2DM) 1. It belongs to the thiazolidinedione class and has been used for treating T2DM since its approval by the US Food and Drug Administration (FDA) in 1999. Additionally, evidence suggests that pioglitazone offers a benefit in reducing the risk of cardiovascular outcomes (e.g., stroke) as well as levels of various inflammatory factors 2, 3. However, in 2005, an increased risk of bladder cancer among patients treated with pioglitazone versus placebo was observed in the prospective pioglitazone clinical trial in macrovascular events (PROactive) 4. Subsequent observational studies seemed to support this finding and indicated a dose‐dependent relationship 5, 6. Given the increased risk of bladder cancer, Germany, France, and then India suspended the marketing of pioglitazone, and the US FDA added a warning against use by patients with active bladder cancer 7.

Subsequently, a number of observational studies were conducted to explore the association between pioglitazone use and risk of bladder cancer, but reached inconsistent conclusions. In 2015, one ten‐year interim analysis in a large observational study of the Kaiser Permanente Northern California (KPNC) database, which was required by US FDA, found that use of pioglitazone was not significantly associated with increased risk of bladder cancer in the United States 8. The null findings were consistent with those of another large retrospective cohort study from four databases in Europe 9, and recently, the 10‐year follow‐up of the PROactive trial also detected no increased risk 10. However, one population‐based cohort study reported a positive association 11. On 12 December 2016, US FDA provided a narrative review of four studies 4, 8, 10, 11 and made a statement of an increased risk of bladder cancer associated with use of pioglitazone 12.

Because of the ongoing safety concerns, there is a critical need for a rigorous assessment of the bladder safety of pioglitazone. Furthermore, several effect modifiers (e.g., gender, region of study, and cumulative dose or duration of pioglitazone) may contribute to the risk of bladder cancer; however, they have not been fully explored in previous meta‐analyses 13, 14, 15, 16, 17, 18. In this systematic review and meta‐analysis, we therefore conducted a cumulative meta‐analysis of all available evidence from observational studies to describe the development of the evidence over time, as well as a dose‐response meta‐analysis to assess this association. Additionally, we assess the potential factors using a meta‐regression analysis.

## Methods

### Search strategy and inclusion criteria

Following the PRISMA guidelines 19, we systematically searched PubMed, Embase, and Cochrane Central Register of Controlled Trials (CENTRAL) to identify randomized controlled trials or observational studies published up to 25 August 2016 that evaluated the association between exposure to pioglitazone and risk of bladder cancer. The combined terms—”((thiazolidinedione* or glitazone* or pioglitazone or rivoglitazone or rosiglitazone or troglitazone) and (neoplasms OR cancer))”—were used with a restriction in “human” only. A manual search was performed on the reference lists of included studies and systematic reviews.

We included randomized controlled trials (RCTs) fulfilling the following inclusion criteria: (1) parallel‐group design; (2) pioglitazone as the intervention versus a control treatment including nonthiazolidinedione therapy, nonpioglitazone therapy, or placebo; and (3) reporting the events of bladder cancer. In addition, we also considered observational studies (cohort or case‐control studies) reporting on the risk of bladder cancer associated with use of pioglitazone versus never use of pioglitazone/thiazolidinedione. We excluded the conference abstracts as well as the articles without reporting the bladder cancer outcome.

### Study selection and data extraction

Two reviewers (HT and WS) independently checked titles and abstracts to exclude articles that clearly did not fulfill the inclusion criteria. Any potentially eligible studies were further assessed by retrieving full texts. A standardized data extraction form was used to collect the following information: study design, drug use, region of study, characteristics of participants, selection criteria, exposure definition, controlled covariates, funding, and data on bladder cancer risk (e.g., adjusted hazard ratio (HR), adjusted risk ratio (RR), and adjusted odds ratio (OR)).

### Risk of bias

Two reviewers (HT and WS) independently used the Cochrane risk‐of‐bias tool and the Newcastle‐Ottawa quality assessment scale (NOS) to assess the quality of RCTs and observational studies, respectively 20, 21. For RCTs, the risk of bias was assessed as low, high, or unclear for each of the following items: random sequence generation (selection bias), allocation concealment (selection bias), blinding (performance bias or detection bias), incomplete outcome data (attrition bias), and selective reporting (reporting bias) 20. For observational studies, a maximum of nine stars were allocated to the domains of selection, comparability, and outcome/exposure, with higher scores indicating better quality 21. Any disagreements were resolved by consensus or referral to a third reviewer (JH).

### Statistical analysis

We pooled the data for RCTs and observational studies separately. For RCTs, a Peto's OR with 95% confidence interval (CI) was used to pool the outcome data due to rare events of bladder cancer 22. For observational studies, OR with 95%CI was pooled using conventional and cumulative random‐effects meta‐analyses. Although the effect measures are different between cohort studies (HR or RR) and case–control studies (OR), they are relative measures and are considered to be similar when the event rate is low (<5%) 23. Statistical heterogeneity was quantified using the *I* ^2^ statistic, with *I* ^2^ of <25%, ≥25% and <75%, and ≥75% indicating low, medium, and high heterogeneity, respectively 24. A meta‐regression analysis was carried out to investigate whether the estimates were affected by the following factors: cumulative dose (≤10.5 g vs. 10.5–28 g vs. >28 g; or ≤14 g vs. 14–40 g vs. >40 g), cumulative duration (≤1 year vs. 1–2 years vs. >2 years; or ≤1.5 year vs. 1.5–4 years vs. >4 years), region of study (Europe vs. United States vs. Asia), type of comparison group (never use of pioglitazone versus never use of thiazolidinedione vs. specified drug), gender (women vs. men), smoking adjusted (adjusted for smoking vs. no adjusted for smoking), and source of funding (industry vs. other). A sensitivity analysis omitting each study successively was performed to investigate the influence of each individual study on the overall meta‐analysis summary estimates. For the studies with possible overlapping patients, additional sensitivity analysis was also performed by including the most recent study only. In addition, publication bias for risk of bladder cancer was assessed using Begg's and Egger's tests, as well as visual inspection of the funnel plots 25.

We tested possible dose and time responses of risk of bladder cancer using restricted cubic splines random‐effects meta‐analysis, with three knots at the 10th, 50th, and 90th percentiles of the distribution. We also used the method described by Greenland and Longnecker 26 and Orsini et al. 27 for the dose‐response analysis to compute the trend from the correlated ORs and 95% CIs across categories of cumulative dose and cumulative duration. The numbers of case and control subjects or person‐years by category were extracted if available. The median or mean cumulative dose or cumulative duration in each category was assigned to the corresponding OR. If median or mean values were unavailable, we used the categorical midpoint. For open‐ended categories, 0.5 times of the higher boundary (for the lowest category) or 1.5 times of the lower boundary (for the highest category) were set to obtain the midpoints 28, 29.

All statistical analyses were performed with STATA (Version 14; Stata Corp., College Station, TX). A two‐tailed *P* < 0.05 was considered statistically significant.

## Results

### Study selection

**Figure 1:**
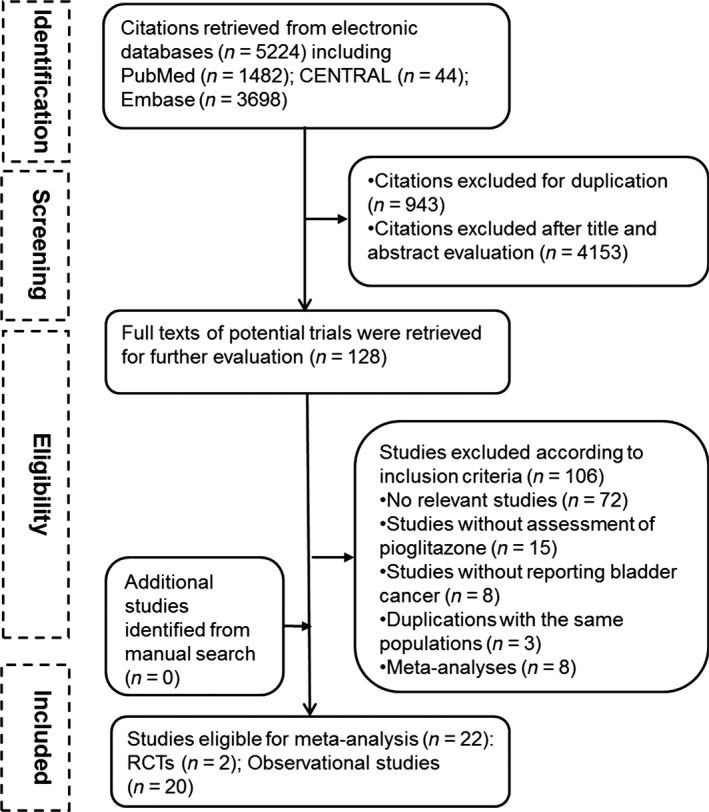
Flowchart of the identification of eligible studies.

Of 5224 citations identified from electronic databases, 128 studies were potentially eligible after initial title and abstract evaluations. After further assessment through retrieving full texts, 22 studies proved eligible, including two RCTs and 20 observational studies (12 cohort studies and eight case–control studies). No additional studies were identified in the manual search. The process of study selection with reasons for exclusion is presented in Figure 1.

### Evidence from RCTs

Two large randomized, placebo‐controlled trials reporting the outcomes of bladder cancer were eligible and included 2, 4. One was performed in patients without diabetes, but had insulin resistance and a recent history of ischemic stroke or transient ischemic attack 2. The other trial included T2DM patients who showed evidence of macrovascular disease 4. The mean length of follow‐up was 4.8 years and 2.9 years, respectively. The risk of bias in both trials was low, and both groups in each trial were generally balanced with respect to demographic and clinical characteristics. The characteristics and risk of bias of included RCTs are presented in Table S1 and Figure S1, respectively**.**

**Figure 2:**
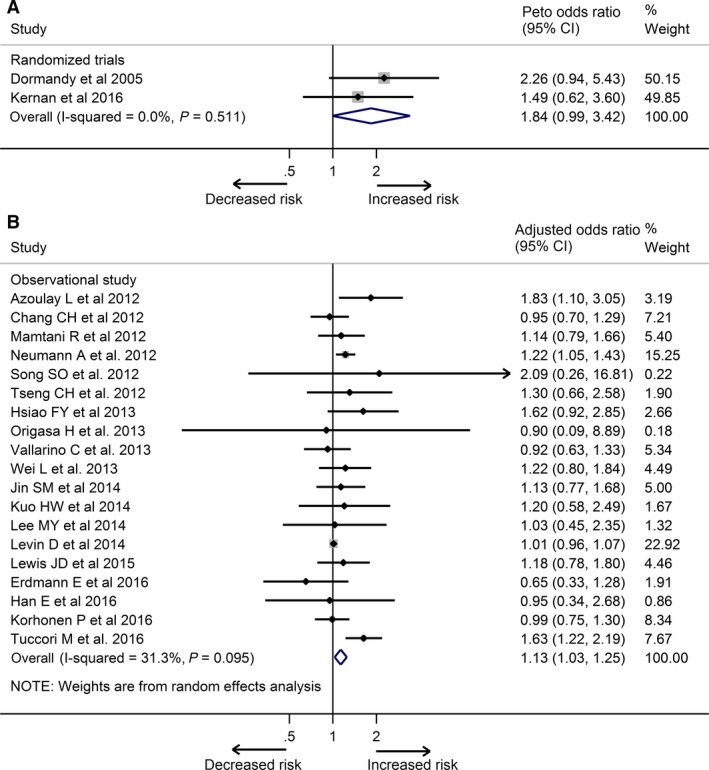
Meta‐analysis of the association between pioglitazone use and risk of bladder cancer based on randomized controlled trials (A) and observational studies (B).

These two trials reported a total of 40 cases of bladder cancer among 9114 patients (raw event rate 0.4%), with 26 cases among 4544 patients (raw event rate 0.6%) treated with pioglitazone and 14 cases among 4570 patients (raw event rate 0.3%) treated with placebo. Meta‐analysis of these two trials showed a borderline increase in the risk of bladder cancer when pioglitazone was compared with placebo (Peto OR, 1.84; 95%CI, 0.99 to 3.42), with no evidence of between‐study heterogeneity (*I* ^2^ = 0%; Fig. 2A). When excluding the cases diagnosed within the first year of exposure to pioglitazone from the PROactive 4, 30, a nonsignificantly increased risk of bladder cancer associated with pioglitazone was observed (Peto OR, 1.63; 95% CI, 0.78 to 3.38).

### Evidence of observational studies

Twenty studies (12 cohort studies and eight case–control studies) involving 4,846,088 individuals reported adjusted estimates of bladder cancer 6, 8, 9, 10, 11, 31, 32, 33, 34, 35, 36, 37, 38, 39, 40, 41, 42, 43, 44, 45 (Table S2). Two studies used the same research database (UK General Practice Research Database) 31, 39, which might lead to overlapping patients. Both studies were included because they provided different information and used different comparison groups (never‐use of pioglitazone vs. never‐use of thiazolidinedione). Five studies published by independent groups were performed in the same databases (Taiwan National Health Insurance) 32, 35, 36, 41, 42. They were all included as they differed in study design, selection of cases and control, and duration of follow‐up, although there may be some overlapping patients. We included one open‐label 10‐year observational follow‐up of a RCT (PROactive), which was considered a cohort study 10. Three studies published by Lewis et al. used the same database 5, 8, 46, so we included the longest follow‐up study 8 and included some data of subgroup from the study published in 2011 5. The included studies were of adequate quality, with more than six stars out of nine in the NOS quality assessment (Table S3). Five observational studies were sponsored by industry (Takeda) 8, 9, 10, 38, 40.

**Figure 3:**
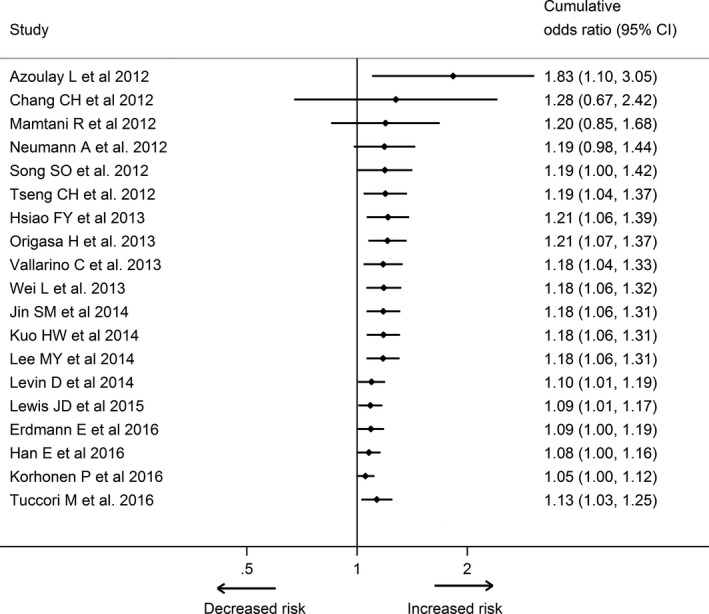
Cumulative meta‐analysis of the association between pioglitazone use and risk of bladder cancer based on adjusted data from observational studies.

Meta‐analysis of adjusted estimates from 19 studies that provided the data of bladder cancer risk showed that ever use of pioglitazone was associated with significantly increased risk of bladder cancer compared with never‐users (OR, 1.13; 95% CI, 1.03 to 1.25), with moderate between‐study heterogeneity (*I* ^2^ = 31.3%; Fig. 2B). Our cumulative meta‐analysis ordered by publication year indicated a significant association between pioglitazone use and never users became evident at the end of 2012 (cumulative OR, 1.19; 95% CI, 1.04 to 1.37). The estimates by cumulative meta‐analysis of subsequent studies resulted in a narrow CI (Fig. 3). Our sensitivity analysis by omitting each study successively showed that our results did not change evidently even if we excluded the most influential study by Tuccori et al. 11 (OR, 1.05; 95% CI, 1.00 to 1.12; Fig. S2). The results also remained stable in the additional sensitivity analysis only including the study performed by Wei et al. 39 using UK General Practice Research Database and the study performed by Lee et al. 42 using Taiwan National Health Insurance (OR, 1.11; 95% CI, 1.00 to 1.23; Fig. S3). There was no evidence of substantial publication bias based on Egger's test (*P *=* *0.09), Begg's test (*P *=* *0.97), or visual inspection of the funnel plot (Fig. S4).

The results from stratified analyses are presented in Table 1. Our meta‐analysis of nine studies that provided the stratified results by cumulative duration showed that, compared with never‐users, pioglitazone use was associated with a significantly higher risk of bladder cancer in the longest cumulative duration exposure (>2 years: OR, 1.49; 95% CI, 1.21 to 1.84) and in the moderate cumulative duration exposure (1–2 years: OR, 1.25; 95% CI, 1.11 to 1.40), but not in the shortest cumulative duration exposure (≤1 year: OR, 1.07; 95% CI, 0.92 to 1.23). Similarly, the meta‐analysis of eight observational studies that provided the stratified results by cumulative dose showed that ever use of pioglitazone was associated with a significantly increased risk of bladder cancer in both the highest cumulative dose exposure (>28 g: OR, 1.66; 95% CI, 1.32 to 2.07) and the moderate cumulative dose exposure (10.5–28 g: OR, 1.27; 95% CI, 1.05 to 1.54), but not in the lowest cumulative dose exposure (<10.5 g: OR, 1.17; 95% CI, 0.99 to 1.39). There is some evidence that the increased risk of bladder cancer associated with pioglitazone showed a time–response relationship (*P *=* *0.01) and dose–response relationship (*P *=* *0.04).

**Table 1:** Stratified analysis of the association between pioglitazone use and risk of bladder cancer based on adjusted data from observational studies

	Number of studies	Odds ratio (95%CI)	*I* ^2^ (%)	*P* for heterogeneity
Overall	19	1.13 (1.03, 1.25)	31.3	–
Smoking adjusted				
Yes	6	1.28 (1.02, 1.61)	43.1	0.07
No	13	1.03 (0.98, 1.08)	0	
Region of study				
Europe	8	1.17 (1.00, 1.36)	67.6	0.77
United States	9	1.03 (0.78, 1.36)	0	
Asia	2	1.11 (0.92, 1.34)	0	
Source of funding				
Industry (Takeda)	5	1.00 (0.85, 1.19)	0	0.17
Other	14	1.20 (1.05, 1.36)	43.8	
Type of comparators				
Never use of Thiazolidinedione	3	1.62 (1.27, 2.08)	0	0.43
Never use of Pioglitazone	13	1.04 (0.99, 1.09)	0	
Rosiglitazone	1	1.14 (0.79, 1.65)	–	
Insulin	1	0.92 (0.63, 1.34)	–	
Placebo	1	0.65 (0.33, 1.28)	–	
Design of study				
Cohort studies	12	1.12 (1.00, 1.24)	40.1	0.69
Case–control studies	7	1.21 (0.96, 1.56)	7.5	
Sex				
Men	3	1.12 (0.96, 1.31)	67.3	0.47
Women	3	1.01 (0.98, 1.08)	0	
Cumulative dose‐1^a^				
≤10.5 g	6	1.17 (0.99, 1.39)	0	0.04
10.5–28 g	4	1.27 (1.05, 1.54)	0	
>28 g	4	1.66 (1.32, 2.07)	0	
Cumulative dose‐2^a^				
≤14 g	2	0.96 (0.79, 1.17)	0	0.80
14–40 g	2	1.07 (0.86, 1.32)	0	
>40 g	2	0.92 (0.58, 1.44)	43.5	
Cumulative duration‐1^a^				
≤1 years	9	1.07 (0.92, 1.23)	33.9	0.01
1–2 years	7	1.25 (1.11, 1.40)	0.2	
>2 years	7	1.49 (1.21, 1.84)	57.5	
Cumulative duration‐2^a^				
≤1.5 years	2	0.97 (0.78, 2.82)	0	0.53
1.5–4 years	2	0.94 (0.73, 1.22)	20.5	
>4 years	2	1.11 (0.85, 1.44)	0	

a Some studies provide the results by cumulative dose (≤10.5 g vs. 10.5–28 g vs. >28 g;) or cumulative duration (≤1 year vs. 1–2 years vs. >2 years), while other studies provide the data based on cumulative dose (≤1.5 year vs. 1.5–4 years vs. >4 years) or cumulative duration (≤14 g vs. 14–40 g vs. >40 g).

The stratified analysis showed that the increased risk of bladder cancer varied depending on the populations from Europe (OR, 1.17; 95% CI, 1.00 to 1.36), Asia (OR, 1.11, 95% CI, 0.92 to 1.34), or United States (OR, 1.03; 95% CI, 0.92 to 1.39), but not significant difference among these three groups (*P *=* *0.77). Similarly, there was little evidence that the risks differed depending on whether pioglitazone compared with never use of any thiazolidinedione (OR, 1.62; 95%CI, 1.27 to 2.08), never use of pioglitazone (OR, 1.04, 95% CI, 0.99 to 1.09), or other comparison groups. An increased risk of bladder cancer was observed in the studies with smoking adjusted (OR, 1.28; 95%CI, 1.02 to 1.61), studies without receiving the funding from industry (OR, 1.20; 95%CI, 1.05 to 1.36), and cohort studies (OR, 1.21; 95%CI, 1.00 to 1.24). There was no significant difference between men and women (*P *=* *0.47).

**Figure 4:**
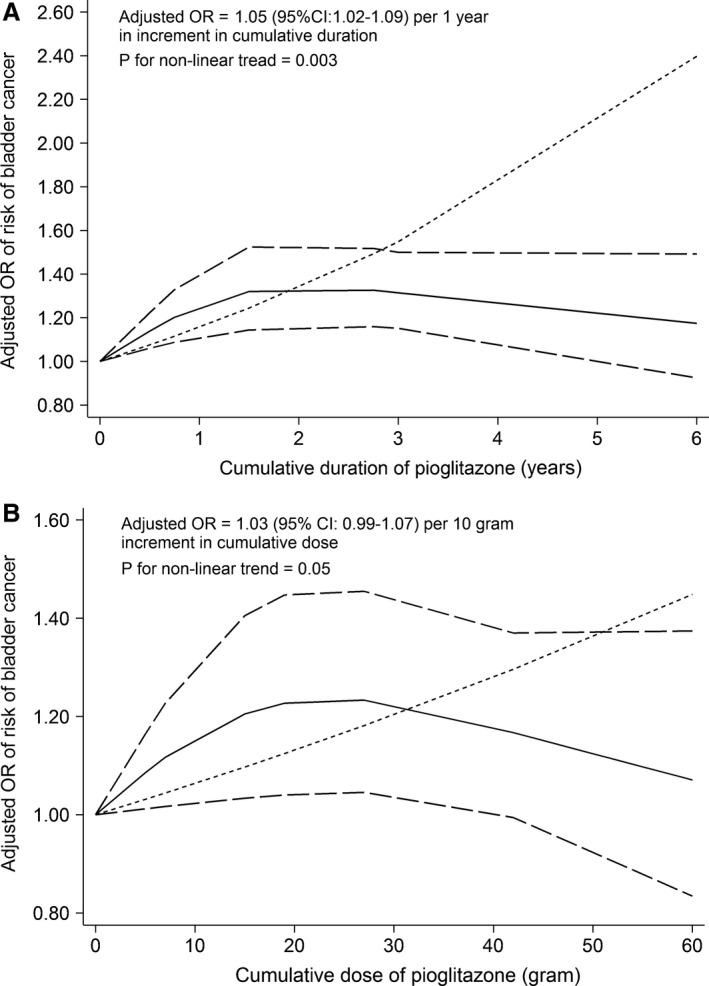
Dose–response relationship between cumulative duration (A) and cumulative dose (B) and risk of bladder cancer in a restricted cubic spline random‐effects meta‐analysis based on adjusted data from observational studies. Note: The solid lines and the long dash lines represent the estimated odds ratios and their corresponding 95% confidence intervals of non‐linear model, respectively. The short dash lines represent estimated odds ratios of linear model.

In addition, from seven studies 5, 8, 9, 11, 31, 35, 42, we found that every 1 year increase in cumulative duration was associated with a 5% higher risk of bladder cancer (OR, 1.05; 95% CI, 1.02 to 1.09) in the cumulative duration between 0 year and 2 years and every 10 gram increase in cumulative dose was associated with a 2% higher risk (OR, 1.03; 95% CI, 0.99 to 1.07) in the cumulative dose between 0 g and 30 g. A restricted cubic splines model revealed a significant non‐linear association between cumulative time and risk of bladder cancer (*P* for non‐linear trend = 0.003, Fig. 4A). Likewise, cumulative dose slightly increased the risk (*P* for non‐linear trend = 0.05, Fig. 4B).

## Discussion

In this study, we analyzed recent evidence to assess the association between pioglitazone and the risk of bladder cancer. Our cumulative meta‐analysis of evidence from observational studies showed that pioglitazone was associated with significantly increased risk of bladder cancer from 2012 onwards and showed borderline increased risk when pooling estimate of RCTs. Furthermore, our meta‐analysis showed that pioglitazone was associated with bladder cancer in both time‐ and dose‐response manner. The association between increased risk of bladder cancer and pioglitazone differed depending on the regions and the choices of comparison groups. Moreover, the increased risk of bladder cancer was observed in the studies which adjusted the smoking status and the studies without supporting by industry. Our sensitivity analysis indicated that the significant association was robust.

Concern persists about the potential risk of bladder cancer associated with pioglitazone. Our meta‐analysis indicated that pioglitazone was associated with a slightly but significantly increased risk of bladder cancer, and this evidence has been stable since 2012. However, the underlying mechanisms have not been fully elucidated. Pioglitazone exerts its anti‐hyperglycemic effects through activation of PPAR *γ* 1, which is highly expressed not only in adipocytes but also in several other tissues (e.g., urinary bladder) 47. Furthermore, PPAR *γ* is also expressed on cancer cells 48. It has been reported that activation of PPAR *γ* may alter tumor growth and progression in nonadipose cells 49. Preclinical research in rats showed that high doses of pioglitazone for 2 years increased risk of bladder tumors among male rats but not among female rats 50. Recently, one study performed on 120 mice showed that prolonged use of pioglitazone might induce significant abnormalities in several biomarkers and hematological indices associated with histopathological changes in the bladder, depending on dose 51. In accordance with the findings from animal studies, our meta‐analysis indicated that greater dose or longer duration of pioglitazone was associated with higher risk of bladder cancer, which supported the notion that pioglitazone's risk of bladder cancer is dose‐dependent, in terms of cumulative dose or cumulative duration. We also noted this risk in the studies with adjusting for the smoking status, as smoking is an important risk factor for bladder cancer 52. Furthermore, we found a significantly increased risk of bladder cancer in the studies without receiving industry funding, which is in contrast to the nulling findings from the studies supported by pharmaceutical company (Takeda). Lexchin et al. 53 reported that the studies sponsored by pharmaceutical companies were more likely to have outcomes favoring the sponsor than were studies with other sponsors.

However, it should be noted that 11 cases of bladder cancer (eight in pioglitazone group and three in the placebo group) from PROactive were diagnosed within the first year of treatment 4, 30. A nonsignificantly increased risk of bladder cancer associated with pioglitazone was observed when excluding those cases within the first year of exposure. Furthermore, some analyses did not suggest a strong dose‐dependence relationship. Several factors in patients with long duration of diabetes might also contribute to the increased risk of bladder cancer. In addition, two large observational studies sponsored by Takeda did not detect any increased risk of bladder cancer among patients with exposure to pioglitazone in terms of dose (≤14 g vs. 14–40 g vs. >40 g) or duration (≤1.5 years vs. 1.5–4 years vs. >4 years) 8, 9. Pioglitazone is generally a second‐ or third‐line oral antidiabetic drug, which suggests that patients treated with pioglitazone may be elderly, who are more likely to have diabetes for a longer time or with higher incidence of comorbidities and complications 54. These factors might lead to the increased risk of bladder cancer 55. Diabetic patients have an increased risk of bladder cancer, especially in those with the duration <5 years 56. In addition, the risk of bladder cancer might be influenced by the use of other antidiabetic drugs. Metformin may prevent, but insulin might promote some cancers 57, 58. Our results indicated that different choices of comparison groups might lead to different assessments of risk. Gender was also considered as a factor that might influence the risk of bladder cancer 56, 59. One cohort study performed in France showed a significant association between pioglitazone and bladder cancer among men but not in women 6. However, our meta‐analysis did not indicate significant gender difference. These factors require further investigation.

Interestingly, our results indicate that the increased risk of bladder cancer associated with pioglitazone was detected in European populations, but not in US or Asian populations. Differences among ethnicities might be involved. A higher rate of bladder cancer was observed in Caucasians when compared with Blacks and Asians 60. European populations primarily consist of white subjects, while those from Asia of course consist of Asian patients. In the United States, the race among different ethnicities differs markedly, with Caucasians having the highest rate. The differences in risk of bladder cancer among these populations could be explained by dissimilar genetic backgrounds, or possibly by the dietary, socioeconomic, and cultural differences among ethnicities 52.

Six meta‐analyses were conducted to evaluate the association between pioglitazone and risk of bladder cancer (Table S4) 13, 14, 15, 16, 17, 18. These meta‐analyses were published between 2012 and 2014 with the latest search time in July 2013. All found that pioglitazone was associated with significantly increased risk of bladder cancer, of which two detected a dose‐response relationship and others identified a duration‐response relationship. Compared with these studies, our meta‐analysis has data updated as of August 2016 and included two large randomized trials and 20 observational studies, of which one large trial and several large observational studies were published since July 2013. We provide more substantial evidence of an association between increased risk of bladder cancer and pioglitazone use in European populations and suggest the choices of comparison groups might influence the results. Our findings also indicated some evidence of bias induced by pharmaceutical companies. In addition, our cumulative and sensitivity meta‐analysis indicated that the positive association was robust.

Our study has several strengths. First, we systematically searched electronic databases and included both randomized trials and observational studies to evaluate the association between pioglitazone and risk of bladder cancer. Second, we explored sources of heterogeneity by performing meta‐regression meta‐analysis and several prespecified stratified analyses. Third, we assessed the robustness of evidence by carrying out cumulative and sensitivity analyses. Finally, a restricted spline regression analysis was used to examine the dose‐response relationship with a generalized least‐squares trend test. We also acknowledge that our meta‐analysis has several limitations. First, our results should be interpreted with caution due to between‐study heterogeneity in exposure and population. The duration of follow‐up, duration of diabetes, and other drugs used varied across studies, although several prespecified stratified analyses were performed to minimize the heterogeneity. The cumulative dose and cumulative duration of pioglitazone use were different. Furthermore, not every study included these results for inclusion in our meta‐analysis. Second, different observational studies adjusted for different sets of confounders, although we used the risk estimates that fully adjusted for the covariates and performed a stratified analysis by major factor adjusted (smoking status). Third, several studies performed a nested case–control in a cohort and reported both results 8, 40. We just used the results from the cohort. Finally, rosiglitazone was not assessed in our study because of current studies and meta‐analyses have found no association between its use and bladder cancer 11, 14.

In summary, our meta‐analysis of currently available evidence suggests that pioglitazone is associated with slightly but significantly increased risk of bladder cancer, indicating a time‐ and dose‐response relationship. The risk of bladder cancer associated with pioglitazone is various and depended on ethnicity and choice of comparison group. Our updated and detailed information suggests cautions and closely monitoring signs for bladder cancer in patients with T2DM, who use pioglitazone, especially those requiring higher doses and longer duration of treatment. However, further well‐designed and well‐conducted studies that clearly define study population and exposure, and adequately adjust confounders are needed to confirm our findings.

## Conflict of Interest

The authors declare that they have no conflict of interest.

## Supporting information


**Table S1.** Characteristics of included RCTs.
**Table S2.** Characteristics of included observational studies.
**Table S3.** Quality assessment of observational studies.
**Table S4.** Summary results of previous meta‐analyses.
**Figure S1.** Risk of bias assessments of included RCTs.
**Figure S2.** Sensitivity analysis of the association between pioglitazone use and risk of bladder cancer when omitting each study successively based on adjusted data from observational studies.
**Figure S3.** Sensitivity analysis of the association between pioglitazone use and risk of bladder cancer by including the most recent study only based on adjusted data from observational studies.
**Figure S4.** Funnel plot of the association between pioglitazone use and risk of bladder cancer based adjusted data from observational studies.
