# Supplementary material for: Pioglitazone and bladder cancer risk: a systematic review and meta‐analysis
Source: Cancer Med. 2018 Feb 24;7(4):1070–80. doi: 10.1002/cam4.1354 (PMC5911601; doi:10.1002/cam4.1354)

Supporting Information

Table S1. Characteristics of included RCTs.

Table S2. Characteristics of included observational studies.

Table S3. Quality assessment of observational studies

Table S4. Summary results of previous meta-analyses

Figure S1. Risk of bias assessments of included RCTs.

Figure S2. Sensitivity analysis of the association between pioglitazone use and risk of bladder cancer when omitting each study successively based on adjusted data from observational studies.

Figure S3. Sensitivity analysis of the association between pioglitazone use and risk of bladder cancer by including the most recent study only based on adjusted data from observational studies.

Figure S4. Funnel plot of the association between pioglitazone use and risk of bladder cancer based adjusted data from observational studies.

Table S1. Characteristics of included randomized controlled trials.

Study	NCT number	Intervention	No. of patients	Patients	Mean Age (years)	Male (%)	Race (Primary)	Mean HbA1c (%)	Mean BMI (kg/m2)	Duration (years)	Sponsored by Takeda
Dormandy et al 2005 [4]	NCT00174993	Pioglitazone versus placebo	5238	Type 2 diabetes patients (35-75 years) who had evidence of macrovascular disease	61.7	66.1	White	7.8	30.9	2.9	Yes
Kernan et al 2016 [2]	NCT00091949	Pioglitazone versus placebo	3876	Patients (≥40 years) without diabetes who had insulin resistance along with a recent history of ischemic stroke or transient ischemic attack	63.5	65.5	White	5.8	30	4.8	Yes

Table S2. Characteristics of included observational studies.

Study	Study design and data source and period	Mean Age (years)	Male (%)	No. of participants	Selection criteria	Exposure definition	Non-exposure definition	Outcome definition	Follow-up (years)	Controlled covariates	Sponsored by Takeda
Azoulay L et al 2012 [31]	Nested case-control analysis; UK general practice research database1988 to 2009;UK	68.9	81.4	115,727 T2DM patients	T2DM patients; newly diagnosed bladder cancer and 20 matched controls	Ever use of pioglitazone	Never use of any TZD	Bladder cancer (medical record)	4.8	Excessive alcohol use, obesity, smoking status, HbA1c, previous bladder conditions, previous cancer, Charlson comorbidity score, and ever use of other antidiabetic agents	No
Chang CH et al 2012 [32]	Nested case-control study; NHIRD; 2000-2007; Taiwan	71	67	7,891 T2DM patients diagnosed in 2000 (1,583 cases and 6,308 controls)	T2DM patients (≥30 years)	Ever use of pioglitazone	No use of pioglitazone	Bladder cancer (ICD-9)	7.9	Pioglitazone, rosiglitazone, short-acting human insulin, metformin, sulfonylurea , number of oral antidiabetic agents, chronic liver disease, statins, aspirin, beta-blockers, chronic kidney dis ease, glinides, nephropathy, cerebrovascular disease, calcium channel blockers, cardiovascular disease, chronic lung disease.	No
Mamtani R et al 2012 [33]	Retrospective cohort study; THIN database; 2000-2010; UK	median 60	57	28,514 patients (pioglitazone: 10,900; rosiglitazone: 17,614)	T2DM patients treated with TZD or SU	Pioglitazone	Rosiglitazone	Bladder cancer (The Read Codes)	3.7	Age, sex, smoking, and hemoglobin HbA1c level	No

Neumann A et al. 2012 [6]	Cohort study; French national health insurance information system; 2006-2009; France	47%> 65	53	1,491,060 patients (pioglitazone: 155,535; No pioglitazone:1,335,525)	T2DM patients aged 40 to 79 years who filled a prescription for a glucose-lowering drug in 2006	Pioglitazone	No exposure to pioglitazone	Bladder cancer (ICD-10)	Up to 3.5	Age, sex, and exposure to glucose-lowering drugs	No
Song SO et al. 2012 [34]	Case-control study; Severance Hospital; 2005 to 2011; Korea	69	84.2	985 patients (bladder cases:329; controlled : 658)	T2DM patients (≥20 with bladder confirmed by cytology	Ever use of pioglitazone	No use of pioglitazone	Bladder cancer confirmed by cytology	NR	NR	No
Tseng CH et al. 2012 [35]	Cohort study; NHIRD;2006 to 2009;Taiwan	NR	NR	54,928 patients from the randomly selected individuals (pioglitazone 2,545; No pioglitazone 52383)	T2DM patients under therapy with oral antidiabetic agents or insulin	Ever prescribed pioglitazone	Never prescribed pioglitazone	bladder cancer (ICD-9)	Up to 4	Age, sex, diabetes duration, nephropathy, urinary tract disease, hypertension, chronic obstructive pulmonary disease, cerebrovascular disease, ischemic heart disease, peripheral arterial disease, eye disease, dyslipidemia, heart failure, rosiglitazone, sulfonylurea, meglitinide, metformin, acarbose, insulin, statin, fibrate, ACE inhibitor/angiotensin receptor blocker, calcium channel blocker, region of residence, occupation, and other cancer before baseline	No

Hsiao FY et al 2013 [36]	Nested case control study; NHIRD;1997-2008; Taiwan	66	68	20,472 patients (bladder cases:3,412; controls:17,060)	T2DM patients with new diagnosed bladder cancer and 5 matched controls	Exposed to pioglitazone	No exposed to pioglitazone	bladder cancer(ICD-9)	3.6	Duration of diabetes, co-morbid conditions, and concomitant medications	No
Origasa H et al. 2013 [37]	Nested case-control study; Tbyama University Hospital Database; 2005 to 2011; Japan	69	74	95 matched patients (40 cases and 55 controls)	T2DM patients with pathologically diagnosed bladder cancer, and selectively matched controlled	Ever use of pioglitazone	Never use of pioglitazone	bladder cancer (pathologically diagnosed)	NR	Age, HbA1c, and other antidiabetic medications	No
Vallarino C et al. 2013 [38]	Retrospective cohort study; i3 InVision Data MartTM database; 2000 to 2010; US	59	57	56,536 (pioglitazone : 38,588; insulin: 17,948	T2DM patients (≥45 years) who were new users of either pioglitazone or insulin	Pioglitazone	Insulin	bladder cancer (ICD-9)	Pioglitazone: 1.9 Insulin: 1.9	Age, gender, drug initiation, medical conditions, and drug use	Yes
Wei L et al. 2013 [39]	Cohort study; GPRD; 2001 to 2010; UK	62	57	34,498 patients (17,249 in each group)	T2DM patients (≥40 years) entered the study at the date of first	Pioglitazone	No use of pioglitazone	bladder cancer (medical record)	Pioglitazone: 3.5; Control:5.3	Age, gender, duration of diabetes, smoking status and body mass index (BMI), insulin treatment and number and type of different oral hypoglycaemic drug classes	No

					prescription for pioglitazone or other oral hypoglycemic drugs during the study period						
Jin SM et al 2014[40]	Retrospective cohort study (nest Case-control) ; four tertiary referral hospitals in Korea; 2005 to 2011; Korea	63	53	113,193 patients (pioglitazone:11,240; controls:101,953)	T2DM patients with two or more clinic visits	Ever use of pioglitazone	never use of pioglitazone	bladder cancer	4.5	Age and sex	Yes
Kuo HW et al 2014 [41]	Nested case control study; NHIRD; 2002 - 2009; Taiwan	70	62	1295 patients randomly selected (bladder cases 259; controls 1036)	T2DM patients with new diagnoses of bladder cancer and 4 randomly selected controls for each case	Pioglitazone	No use of pioglitazone	bladder cancer (ICD-9)	NR	Nephropathy, urinary tract diseases, urinary tract infection, urinary tract stone, hypertension, chronic obstructive pulmonary disease, stroke, ischemic heart disease, peripheral arterial diseases, eye disease, and dyslipidemia.	No
Lee MY et al 2014 [42]	Cohort study; NHI Research Database;2005-2009; Taiwan	>60 years: 66%	47	34,970 diabetes patients with the entry date of 2003 (pioglitazone:3,497;	T2DM	Ever use of pioglitazone	Never use of pioglitazone	Bladder cancer (ICD-9)	4	Sex, age, duration of diabetes, other diabetes medications, income, residential area, nephritis, chronic kidney disease, kidney infections, hydronephrosis, calculus of the lower urinary tract, cystitis, other disorders of the urethra and urinary tract,	No

				never users of pioglitazone:31,473）						hypertension and hyperlipidemia	
Levin D et al 2014 [43]	Cohort study; British Columbia (2000-2004), Finland (2001-2008), Manchester (2001-2007), Rotterdam (2001 - 2004), Scotland (2001 - 2006) and the UK Clinical Practice Research Datalink (2003-2009; Europe	63	53	Scotland: 252,269; CPRD: 156,443; Finland: 426,767; British Columbia: 153.862; Rotterdam: 6,694; Manchester: 11,561	T2DM	Ever exposure to pioglitazone	No exposure to pioglitazone	Bladder cancer (ICD-10)	4.0 to 7.4	Age, calendar year, and ever exposure to pioglitazone	No
Lewis JD et al 2015 [8]	Cohort and nested case-control study; KPNC; 1997-2002 until December 2012; US	>60 years: 49%	54	193,099 patients (pioglitazone:34,181; Never user:158,918)	T2DM patients (≥40 years)	Ever use of pioglitazone	Never use of pioglitazone	Bladder cancer (pathology report)	10	Age, sex, and year of cohort entry, smoking, race/ethnicity, other diabetes medications, other bladder conditions, hemoglobin A1c concentration and the interaction with new diagnosis of diabetes, and duration of diabetes, the 3-level time-updated proteinuria testing, variable median household income, congestive heart failure, cancer other than bladder cancer, renal insufficiency	Yes

Lewis JD et al 2011 [5]	Cohort and nested case-control study; KPNC; 1997-2002 until April 2008; US	>60 years: 49%	54	193,099 patients (pioglitazone:30,173; Never user:162,926)	T2DM patients (≥40 years)	Ever use of pioglitazone	Never use of pioglitazone	Bladder cancer (pathology report)	10	Age, sex, and year of cohort entry, smoking, race/ethnicity, other diabetes medications, other bladder conditions, hemoglobin A1c concentration and the interaction with new diagnosis of diabetes, and duration of diabetes, the 3-level time-updated proteinuria testing, variable median household income, congestive heart failure, cancer other than bladder cancer, renal insufficiency	Yes
Erdmann E et al 2016 [10]	PROactive; randomized open label trial; Europe	63	65	3599 patients	T2DM patients completed the final visit of PROactive	Pioglitazone	Placebo	Bladder cancer	7.8	NR	Yes
Han E et al 2016 [44]	Nested case control study; Korean NHI Service National Sample Cohort; 2002 to 2013; Korea	>60 years: 83%	81.2	935 patients (bladder cancers: 85; controls:850)	T2DM patients with new diagnosed bladder cancer and 10 matched controls	Ever use of pioglitazone	Never use of TZD	Bladder cancer (ICD-10)	NR	Antidiabetic medication, aspirin, statin use, past history of any cancer, renal disease, urolithiasis, other ureter or bladder diseases, congestive heart failure, alcoholic liver disease, Charlson comorbidity score, household income level, and residential area	No
Korhonen P et al 2016 [9]	Retrospective cohort study; Healthcare databases from Finland (1988-2011), the Netherlands(1995-2011), Sweden (2005-2011), and the	64	55	373,446 patients (pioglitazone: 56,337; other diabetes drug treatments: 317,109)	T2DM patients (≥40 years) who initiated diabetic treatment	Ever exposed to pioglitazone	Never exposed to pioglitazone	Bladder cancer (ICD-10)	2.9	Age, sex, diabetic drug treatments, exact matching variables, groups based on quintiles of propensity scores, all variables used in the propensity score, plus possible confounding variables	Yes

	UK (1987-2011); Europe										
Mackenzie TA et al 2016 [45]	Retrospective cohort study; Medicare fee-for-service plan using inpatient, outpatient (2003–2011) and prescription (2006–2011) administrative data; US	75.1	38	Prevalent cohort:1,161,443; Pioglitazone 38,091	T2DM received diabetes medications	Pioglitazone	No use of pioglitazone	Bladder cancer (ICD-9)	3.5	Age, gender, race; low-income subsidy for Medicare Part D, alcohol abuse, chronic obstructive lung disease and/or tobacco use, obesity, diabetes complications, and Charlson comorbidities	No
Tuccori M et al. 2016 [11]	Cohort study; UK CPRD; 2000 to 2013; UK	64	59	145,806 (pioglitazone 921; rosiglitazone 2127; No use 142,758)	T2DM patients (≥40 years) have at least one year of CPRD medical history before first prescription	Pioglitazone	No use of TZD	Bladder cancer (read code classification)	4.7	Age, year of cohort entry, sex, alcohol-related disorders, smoking status, obesity, haemoglobin A1c, previous cancer, bladder conditions, Charlson comorbidity score, duration of treated diabetes, and urine protein testing.	No

T2DM, type 2 diabetes; NIH, National Institutes of Health; CPRD, Clinical Practice Research Datalink; KPNC, Kaiser Permanente Northern California; NHIRD, National Health Insurance Research Database; GPRD, General Practice Research Database; THIN, The Health Improvement Network; TZD, thiazolidinedione; SU, sulfonylureas; ICD, International Classification of Diseases.

Table S3. Quality assessment of observational studies

Study	Selection	Comparability	Exposure/Outcome	Total
Azoulay L et al 2012 [31]	****	**	***	9
Chang CH et al 2012 [32]	****	*	***	8
Mamtani R et al 2012 [33]	****	**	***	9
Neumann A et al. 2012 [16]	****	*	***	8
Song SO et al. 2012 [34]	****		***	7
Tseng CH et al. 2012 [35]	***	*	***	7
Hsiao FY et al 2013 [36]	****	*	***	8
Origasa H et al. 2013 [37]	***	*	**	6
Vallarino C et al. 2013 [38]	****	*	***	8
Wei L et al. 2013 [39]	***	**	***	8
Jin SM et al 2014 [40]	****	*	***	8
Kuo HW et al 2014 [41]	****	*	***	8
Lee MY et al 2014 [42]	***	*	***	7
Levin D et al 2014 [43]	****	*	***	8
Lewis JD et al 2015 [8]	****	**	***	9
Erdmann E et al 2016 [10]	-	-	-	-
Han E et al 2016 [44]	****	*	***	8
Korhonen P et al 2016 [9]	****	*	***	8
Mackenzie TA et al 2016 [45]	**	**	***	7
Tuccori M et al. 2016 [11]	****	**	***	9

Table 4. Summary results of previous meta-analyses

Study	Search time	Interventions	Design of study included (n/N)	Analysis model/ subgroup analysis	Bladder cancer and relevant results
Zhu Z et al, 2012 [13]	January, 2012	Pioglitazone	RCTs and observational studies (5/2,350,908)	Fixed-effects model; Subgroup: cumulative dose or duration	RR 1.17; 95% CI (1.03-1.32); Duration response relationship
Turner RM et al 2014 [14]	July, 2013	Thiazolidinedione (pioglitazone or rosiglitazone)	RCTs (3/7878) Observational studies (8/1,982,536)	Fixed-effects model; Subgroup: cumulative dose or duration	RCT: OR 2.51; 95% CI (1.09-5.80); Observational studies: OR 1.21; 95%CI (1.09-1.35); Dose response relationship
Monami M et al, 2014 [15]	August, 2011	Thiazolidinedione (pioglitazone or rosiglitazone)	RCTs (3/6272)	Fixed-effects model; NR	OR 2.05;95% CI (0.84-5.02)
He SY et al. 2014 [16]	July, 2012	Pioglitazone	Observational studies and RCTs (9/2,596,856)	Fixed-effects model; Subgroup: design, gender, cumulative dose or duration	HR 1.48 95%CI (1.09-2.00); Dose-response relationship
Ferwana M et al. 2013 [17]	October, 2012	Pioglitazone	Observational studies and RCTs (6/215,142)	Random-effects model; Subgroup: cumulative dose or duration	HR 1.23; 95%CI (1.09-1.39); Duration response relationship
Colmers IN et al 2012 [18]	March, 2012	Thiazolidinedione (pioglitazone or rosiglitazone)	RCT(1/5238) Observational study (3/1,739,087)	Random-effects model; NR	RCT: RR 2.36;95%CI (0.91-6.13); Observational studies: RR 1.22; 95% CI (1.07-1.39)
n/N: number of studies/number of patients; RCTs: randomized controlled trials; RR, risk ratio; OR, odds ratio; HR, hazard risk; CI, confidence interval; NA: not reported					

Figure S1. Risk of bias assessments of included randomized controlled trials

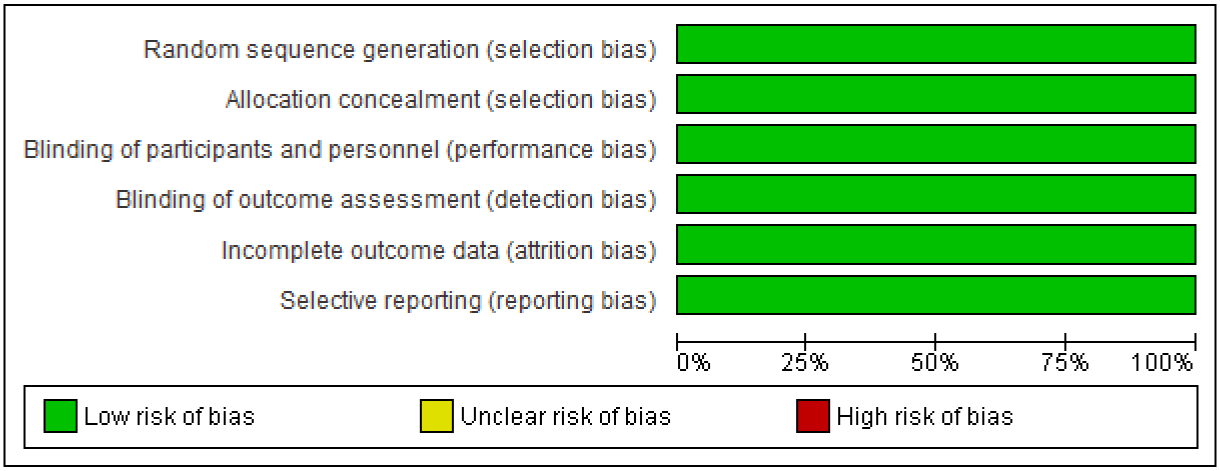

Figure S2. Sensitivity analysis of the association between pioglitazone use and risk of bladder cancer when omitting one study each time based on adjusted data from observational studies

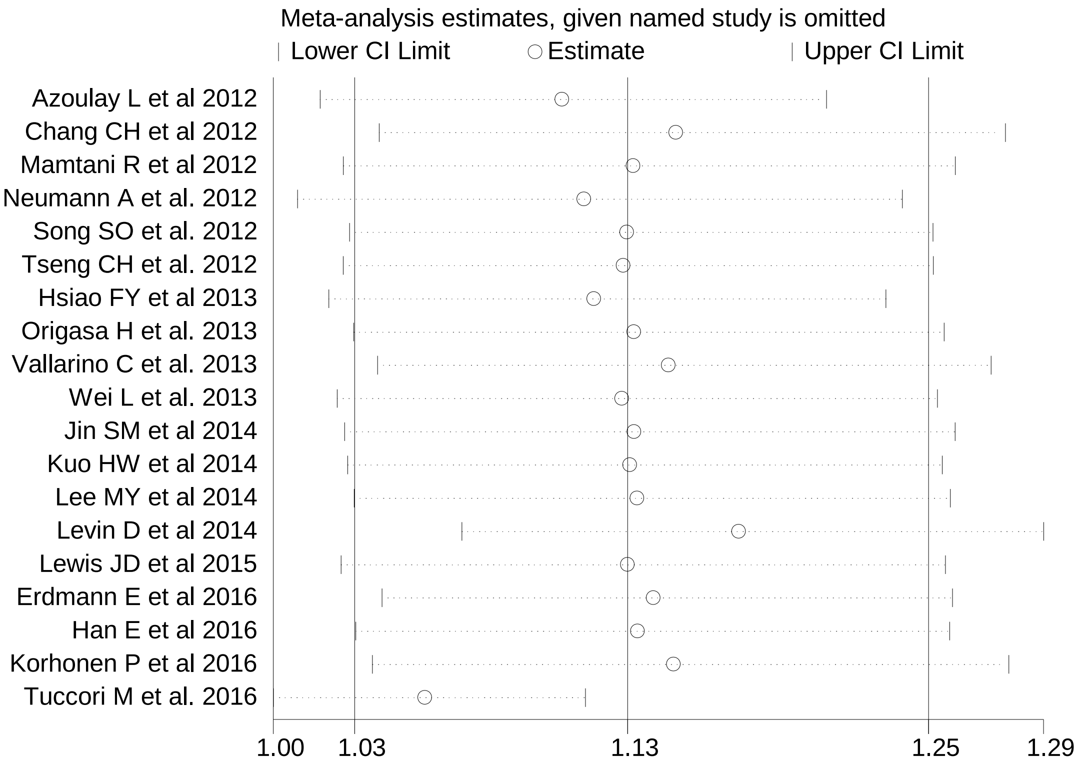

Figure S3. Sensitivity analysis of the association between pioglitazone use and risk of bladder cancer by including the most recent studies based on adjusted data from observational studies. For the studies with possible overlapping patients (Azoulay L et al 2012 and Wei L et al 2013 based on UK General Practice Research Database; Chang CH et al 2012, Kuo HW et al 2014, Lee MY et al 2014, Tseng CH et al 2012, and Hsiao FY et al 2013 based on Taiwan National Health Insurance databases), additional sensitivity analysis was performed by including the most recent study only (Wei L et al 2013 and Lee MY et al 2014)

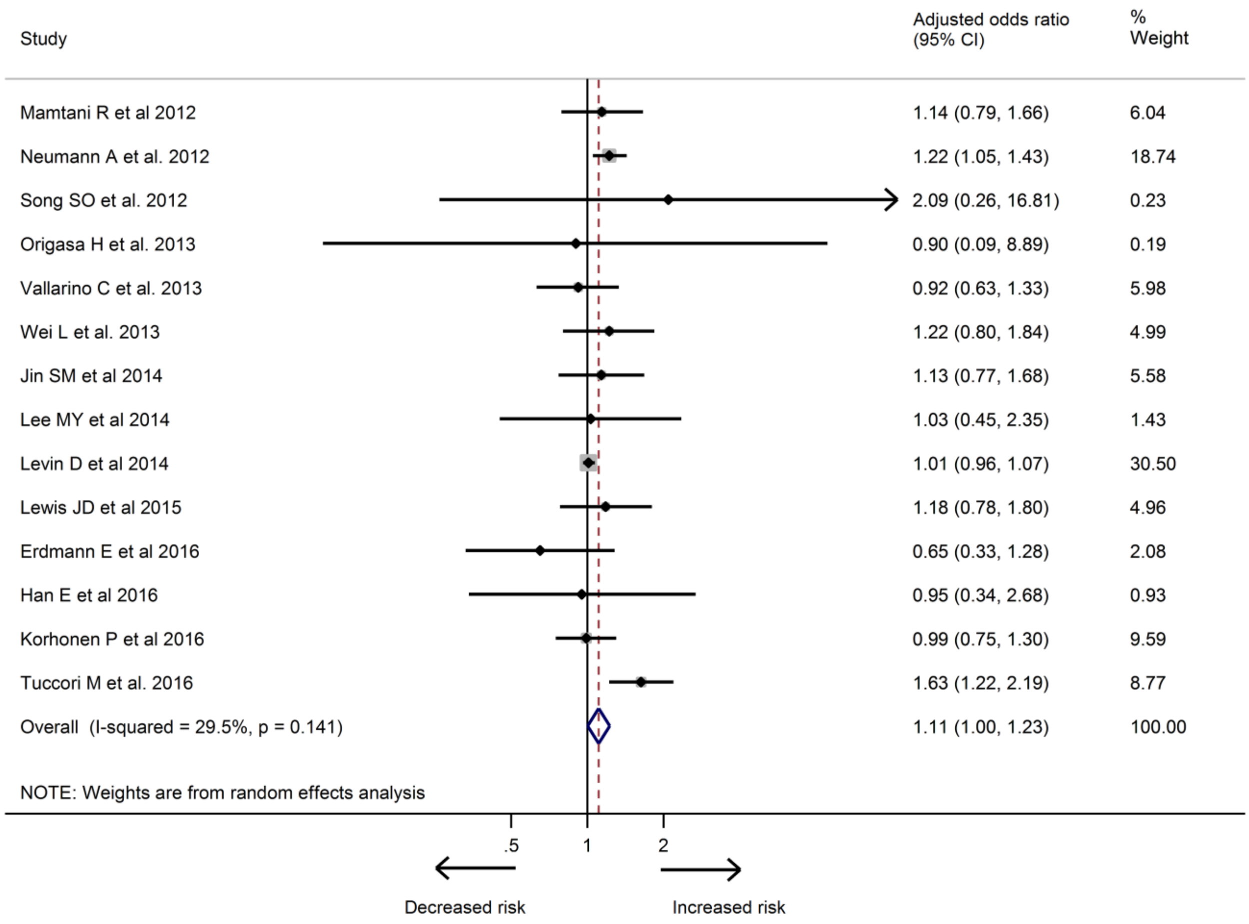

Figure S4. Funnel plot of the association between pioglitazone use and risk of bladder cancer based on adjusted data from observational studies.

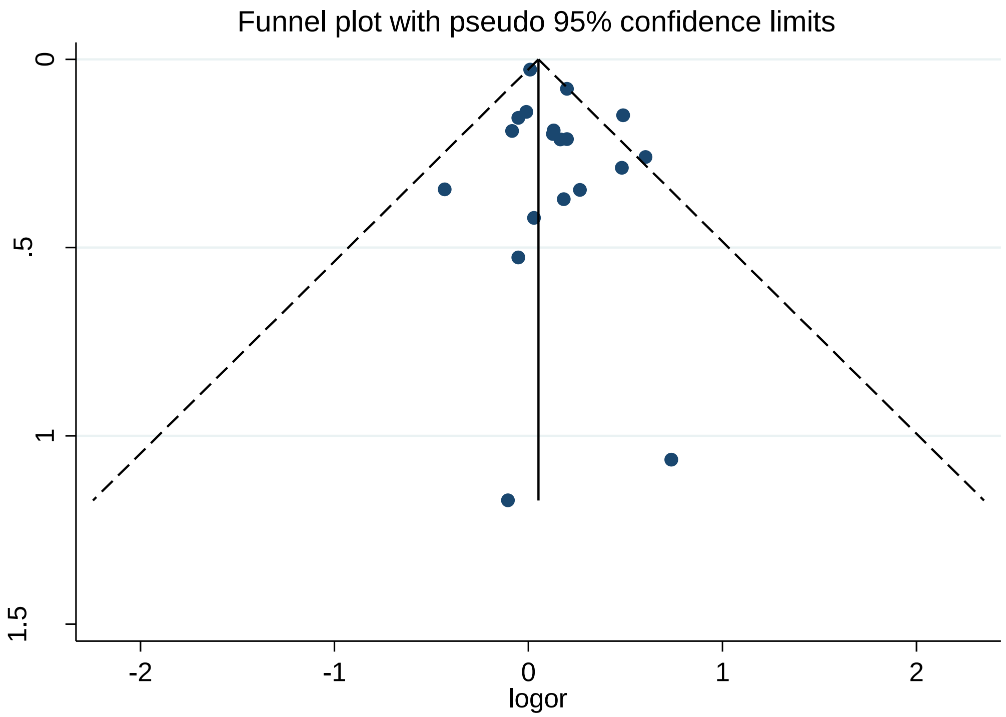

Supplement: Supplementary file 1 — Table S1. Characteristics of included RCTs. Table S2. Characteristics of included observational studies. Table S3. Quality assessment of observational studies. Table S4. Summary results of previous meta‐analyses. Figure S1. Risk of bias assessments of included RCTs. Figure S2. Sensitivity analysis of the association between pioglitazone use and risk of bladder cancer when omitting each study successively based on adjusted data from observational studies. Figure S3. Sensitivity analysis of the association between pioglitazone use and risk of bladder cancer by including the most recent study only based on adjusted data from observational studies. Figure S4. Funnel plot of the association between pioglitazone use and risk of bladder cancer based adjusted data from observational studies. [file CAM4-7-1070-s001.pdf]
